# Perturbation of Lytic and Latent Gammaherpesvirus Infection in the Absence of the Inhibitory Receptor CEACAM1

**DOI:** 10.1371/journal.pone.0006317

**Published:** 2009-07-21

**Authors:** Heiko Adler, Susanne El-Gogo, Simone Guggemoos, Wolfgang Zimmermann, Nicole Beauchemin, Robert Kammerer

**Affiliations:** 1 Helmholtz Zentrum München - National Research Center for Environmental Health, Institute of Molecular Immunology, CCG HCT, Munich, Germany; 2 Institute of Virology, Technical University Munich, Munich, Germany; 3 Tumor Immunology Laboratory, LIFE Center, Klinikum Grosshadern, Ludwig-Maximilians-University, Munich, Germany; 4 Goodman Cancer Centre, McGill University, Montreal, Quebec, Canada; 5 Institute of Immunology, Friedrich-Loeffler-Institut, Tuebingen, Germany; University of Toronto, Canada

## Abstract

Control of gammaherpesvirus infections requires a complex, well orchestrated immune response regulated by positive and negative co-signaling molecules. While the impact of co-stimulatory molecules has been addressed in various studies, the role of co-inhibitory receptors has not been tested. The ITIM-bearing CEACAM1 is an inhibitory receptor expressed by a variety of immune cells, including B, T and NK cells. Using *Ceacam1^−/−^* mice, we analyzed the *in vivo* function of CEACAM1 during acute and latent murine gammaherpesvirus 68 (MHV-68) infection. During acute lytic replication, we observed lower virus titers in the lungs of *Ceacam1^−/−^* mice than in WT mice. In contrast, during latency amplification, *Ceacam1^−/−^* mice displayed increased splenomegaly and a higher latent viral load in the spleen. Analysis of the immune response revealed increased virus-specific antibody levels in *Ceacam1^−/−^* mice, while the magnitude of the T cell-mediated antiviral immune response was reduced. These findings suggest that inhibitory receptors can modulate the efficacy of immune responses against gammaherpesvirus infections.

## Introduction

Murine gammaherpesvirus 68 (MHV-68) is a natural pathogen of wild murine rodents [1], and has recently been established as a mouse model to study gammaherpesvirus pathogenesis [2], [3]. In humans, the prototypic γ1-herpesvirus, Epstein-Barr virus (EBV), is associated with lymphomas and nasopharyngeal carcinoma [4]. Human Herpesvirus-8 (HHV-8, KSHV), a γ2-herpesvirus, is associated with lymphoproliferative disorders and Kaposi's sarcoma [5].

Intranasal infection of mice with MHV-68 results in an acute, productive infection in the lung with viral titers reaching the peak around day 6, and clearance of lytic virus around day 10 to 14 post infection, mainly by CD8^+^ T cells [6], [7]. Latency is established by virus traveling from the lung to the mediastinal lymph nodes. B cells from the mediastinal lymph nodes then traffic to the spleen and other lymphoid organs and establishment of life-long latency takes place [6]. B lymphocytes are the major reservoir harboring latent MHV-68 [8], but macrophages [9], dendritic cells [10] and lung epithelial cells [11] have also been shown to harbor latent virus. Memory B cells are the major reservoir for long term latency [12]–[14]. The establishment of latency in the spleen is associated with a marked splenomegaly and an increase in the number of splenocytes which peaks around 2–3 weeks post-infection. This process is driven by CD4^+^ T cells and depends on MHV-68-infected B cells in the spleen [15]. The splenic mononucleosis is associated with a strong increase in the number of latently infected B cells. Following the amplification of latency, there is both a decrease of the splenomegaly and of the number of latently infected splenocytes back to a basal level [6]. Multiple immune mechanisms including CD8^+^ T cells, CD4^+^ T cells and antibodies contribute to the control of latency and preventing recrudescence of lytic virus [6], [16], [17].

The CEA-related cell adhesion molecule 1 (CEACAM1) is one of the primordial members of the carcinoembryonic antigen (CEA) family, which itself belongs to the immunoglobulin superfamily [18]. This highly glycosylated protein is abundantly expressed in epithelia, vessel endothelia and leukocytes. In most CEACAM1-expressing cell types, the molecule occurs in two major splice variants differing in their cytoplasmic domain, called CEACAM1-L and CEACAM1-S. The ‘L’-isoforms encompass approximately 70 amino acids in the cytoplasmic domain, including tyrosines that are part of an immunoreceptor tyrosine-based inhibitory motif (ITIM) and an immunoreceptor tyrosine-based switch motif (ITSM). After tyrosine phosphorylation, CEACAM1-L can bind SH2 domain-containing proteins, thereby activating Src-family tyrosine kinases [19], [20] and the protein tyrosine phosphatases SHP-1 and SHP-2 [21]. The ‘S’-isoforms encode 10 cytoplasmic residues without ITIM/ITSM motifs. CEACAM1-mediated signals regulate the function of various immune cells. We and others have demonstrated that CEACAM1 can amplify T cell responses under certain conditions [22], [23]. Likewise, targeting CEACAM1 with the mAb AgB10 can enhance murine B cell proliferation through activation of the c-Jun NH2-terminal kinase (JNK) pathway [24]. The same mAb induces maturation and chemokine/cytokine secretion of murine dendritic cells [25]. It was also shown that CEACAM1 can inhibit signals delivered by immunoreceptor tyrosine-based activation motif (ITAM)-containing molecules [26]–[30]. Furthermore, CEACAM1 can inhibit NK cell cytotoxicity when co-ligated with NK cell-activating receptors [31]. These observations indicate that CEACAM1 participates in the regulation of immune responses by delivering signals, and modifying signals transduced by other molecules [32]. Recently, Nagaishi and coworkers demonstrated that *in vivo*, CEACAM1-L overexpression or deletion in T cells resulted in T cell inhibition or activation, respectively [30].

Here, we took advantage of *Ceacam1^−/−^* mice to determine for the first time the outcome of integrated CEACAM1 signaling during the immune response to a naturally occurring gammaherpesvirus infection. Based on the results obtained by functional analyses of CEACAM1 using purified immune cells one would expect an enhanced NK, T and B cell response in *Ceacam1^−/−^* mice, favoring the control of MHV-68 infection. Although *Ceacam1^−/−^* mice displayed an enhanced control of the acute lytic MHV-68 lung infection, the absence of CEACAM1 resulted in elevated viral loads and increased splenomegaly during latency amplification.

## Results

### Lytic virus titers in the lungs are reduced in *Ceacam1^−/−^* mice

**Figure 1:**
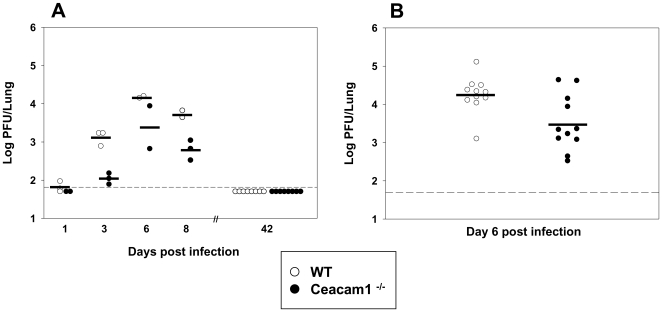
Virus titers in the lung are reduced in *Ceacam1^−/−^* mice. A) In a kinetic experiment, mice were infected i.n. with 5×10^4^ PFU. At days 1, 3, 6, 8 and 42, lungs were harvested and virus titers of lung homogenates were determined by plaque assay on BHK-21 cells. Each symbol represents a single mouse. The horizontal bars indicate the means. The difference between WT mice and *Ceacam1^−/−^* mice was statistically significant (p = 0.001 by two-way ANOVA). B) Mice were infected i.n. with 5×10^4^ PFU. At day 6 after infection, lungs were harvested and virus titers were determined as described in A. The mean viral titer in lungs of *Ceacam1^−/−^* mice (n = 11) was significantly lower than in control mice (n = 11) (p = 0.01). The detection limit of the assay is indicated by the dashed line.

To analyze lytic replication, *Ceacam1^−/−^* and WT mice were infected intranasally (i.n.) with 5×10^4^ PFU. This viral dose has frequently been used by us for i.n. infection and is within the range of doses which have been shown to result in stable levels of acute and latent infection [33]. First, in a kinetic experiment, lungs were harvested at various time points after infection and lytic virus titers in lung homogenates were determined by plaque assay on BHK-21 cells. Lungs of *Ceacam1^−/−^* mice contained significantly less lytic virus when compared to WT mice (Fig. 1A). Next, we analyzed additional animals on day 6 after infection, the time point at which viral titers usually reach a peak. As in the kinetic experiment, lungs of *Ceacam1^−/−^* mice contained significantly less lytic virus when compared to WT mice (Fig. 1B). These data suggested that *Ceacam1^−/−^* mice can control the acute infection more efficiently than WT mice. There was no lytic virus detectable in lung homogenates of both *Ceacam1^−/−^* and WT mice at day 42 after infection, even after *in vitro* amplification (Fig. 1A and data not shown).

### The latent viral load is increased in *Ceacam1^−/−^* mice

**Figure 2:**
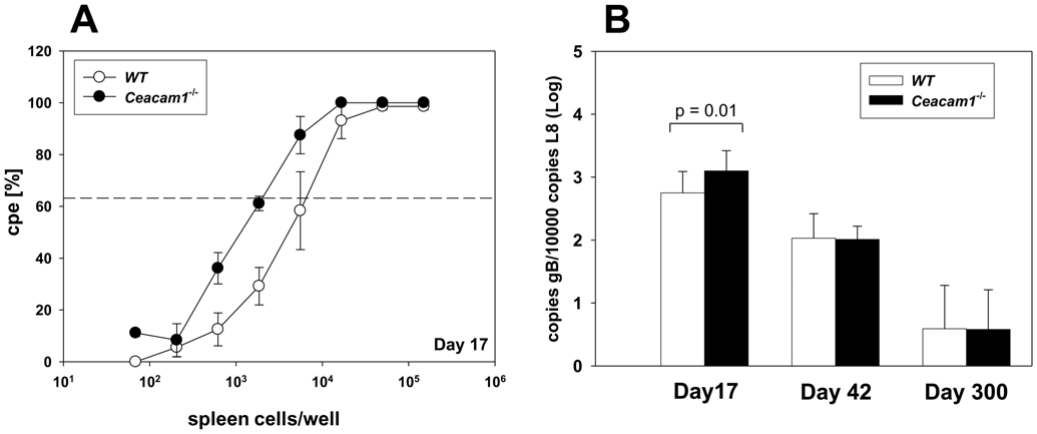
*Ex vivo* virus reactivation and viral genomic load are increased in *Ceacam1^−/−^* mice. A) E*x vivo* reactivation of splenocytes. Mice were infected i.n. with 5×10^4^ PFU of MHV-68. At the indicated time points after infection, spleens were harvested and single splenocyte suspensions were prepared and analyzed in the *ex vivo* reactivation assay. Data shown in panel A are means±SEM pooled from three independent experiments. In each experiment, splenocytes from 3 to 5 mice per group were pooled. The dashed line in panel A indicates the point of 63.2 % Poisson distribution, determined by nonlinear regression, which was used to calculate the frequency of cells reactivating lytic MHV-68 replication. To calculate significance, frequencies of reactivation events were statistically analyzed by paired t-test over all cell dilutions. The statistical significance of the difference between WT and *Ceacam1^−/−^* mice is p = 0.02. B) Viral genomic load. Mice were infected i.n. with 5×10^4^ PFU of MHV-68. At days 17, 42 and 300 after infection, spleens were harvested, single splenocyte suspensions were prepared and used for DNA isolation for real time PCR analysis. The data are presented as viral genome copy numbers (as determined by quantitation of the copy number of the MHV-68 gB gene) relative to the copy number of the murine ribosomal protein L8 gene (*Rpl8)*. Data shown are means±SD of 14 mice per group at day 17, 8 mice per group at day 42 and 2–3 mice per group at day 300.

The establishment of latency in the spleen is associated with an increase in the number of latently infected B cells which peaks around 2–3 weeks after infection. To analyze the latent phase of infection, mice were infected i.n. with 5×10^4^ PFU. The number of latently infected splenocytes can be determined by measuring the number of reactivating splenocytes in the ex vivo reactivation assay and by evaluating the viral genomic load by quantitative PCR. Thus, we determined the extent of *ex vivo* reactivation of splenocytes at day 17 after infection. The frequency of *ex vivo* reactivation of splenocytes isolated from *Ceacam1^−/−^* mice (1 in 1938) was significantly higher than in WT mice (1 in 6453; Fig. 2A). Consistent with the data from the *ex vivo* reactivation assay, the viral genomic load, as determined by quantitative PCR, was significantly higher in *Ceacam1^−/−^* mice (164 copies of viral gB gene per 1000 copies of the ribosomal protein gene L8) as compared to WT mice (82 copies gB per 1000 copies L8) at day 17 after infection (Fig. 2B). At days 42 and 300 after infection, the viral genomic load was not significantly different between the two groups. These data demonstrated a higher number of latently infected cells in *Ceacam1^−/−^* mice when compared to WT mice.

### CEACAM1 expression on splenic B and T cells changes only marginally during infection

**Figure 3:**
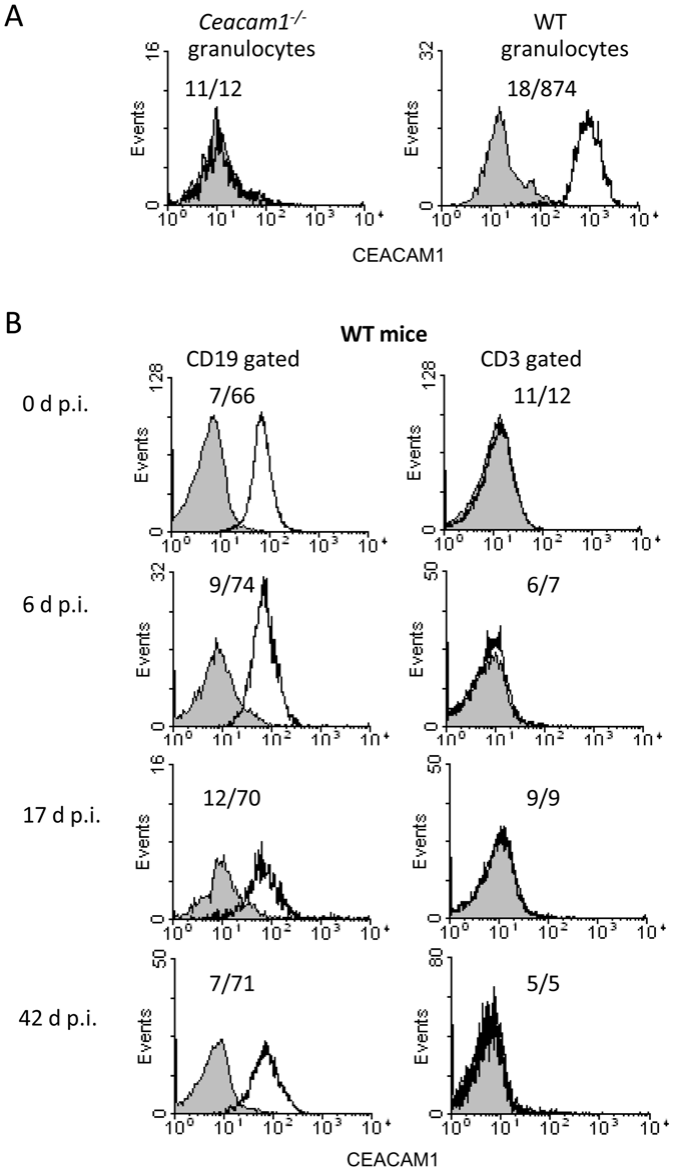
CEACAM1 expression on splenic B and T cells changes only marginally during infection. Mice were infected i.n. with 5×10^4^ PFU of MHV-68. At days 0, 6, 17 and 42 after infection, CEACAM1 expression by spleen cells was determined by three color flow cytometry. The indicated cells were labeled either with the CEACAM1-specific mAb CC1 (open curves) or an isotype-matched antibody (filled curves). A) Granulocytes from *Ceacam1^−/−^* and WT mice were gated according to their characteristic forward/sideward scatter (verified by anti-GR1 (Ly6G) staining) and analysed for CEACAM1 expression. B) CEACAM1 expression on lymphocyte subpopulations from WT mice at different time points after infection. Cells were gated for expression of CD19 (B cells; left panel) and for expression of CD3 (T cells; right panel). The mean fluorescence intensity (mfi) is indicated by numbers (mfi isotype control/mfi specific staining). The experiment was repeated three times with similar results.

As expected, strong expression of CEACAM1 was observed on granulocytes of WT mice but not of *Ceacam1^−/−^* mice (Fig. 3A). In WT mice, CEACAM1 was also strongly expressed on B cells while it was undetectable on T cells. CEACAM1 expression was unaltered on different leukocyte populations during MHV-68 infection (Fig. 3B and data not shown), with the exception of a marginal downregulation of CEACAM1 on B cells at day 17 p.i. (Fig. 3B).

### Increased splenomegaly and unique changes of spleen cell composition during MHV-68 infection of *Ceacam1^−/−^* mice

**Figure 4:**
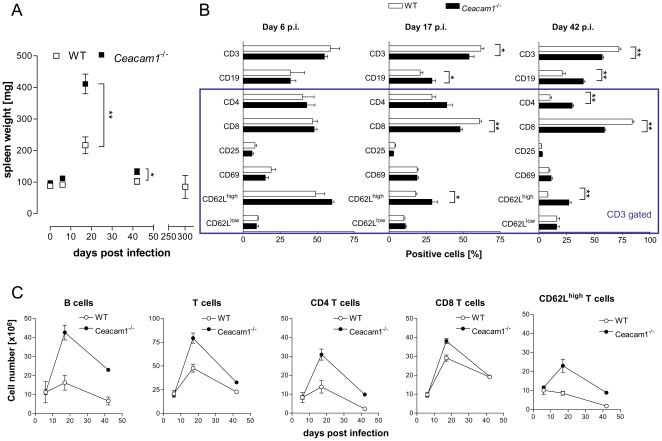
Splenomegaly and composition of spleen cells during MHV-68 infection differ in *Ceacam1^−/−^* and WT mice. A) Splenomegaly. Mice were infected i.n. with 5×10^4^ PFU of MHV-68. At the indicated time points after infection, spleen weights were determined. Data shown in panel A are means±SD of 5 mice (day 6), 11 mice (day 17), 8 mice (day 42) and 3 mice (day 300). Data from uninfected (naive) mice are shown for comparison (n = 24 for WT and n = 13 for *Ceacam1^−/−^* mice). B) Mice were infected i.n. with 5×10^4^ PFU of MHV-68. At days 6, 17 and 42 after infection, the immunophenotype of spleen cells was determined by three color flow cytometry. In each experiment, three mice were used per group, and the experiments were repeated twice (day 6 p.i.) and three times (days 17 and 42 p.i.), respectively. For determination of the percentage of CD3 and CD19 positive cells, only cells within the lymphocyte forward/sideward scatter gate were accepted. For all other markers (indicated by the blue box), only CD3^+^ cells were accepted. CD62L^medium^ cells are not depicted. Asterisks denote statistically significant differences between WT and *Ceacam1^−/−^* mice (*: p < 0.05; **: p < 0.01). C) The absolute cell numbers were calculated as follows: number of cells = number of lymphocytes per spleen×living cells (%) / 100.

The establishment of latency in the spleen is associated with a marked splenomegaly which peaks around 2–3 weeks after infection. Thus, we first determined the extent of splenomegaly at different time points after infection. Both at day 17 and 42 after infection, *Ceacam1^−/−^* mice showed a significantly higher spleen weight than WT mice (Fig. 4A). Similar to the spleen weight, the total number of spleen cells was significantly higher in *Ceacam1^−/−^* mice than in WT mice at day 17 (1.94 fold) and at day 42 (1.90 fold) after infection (data not shown). Second, we characterized the lymphocyte subpopulations in the spleen at days 6, 17 and 42 p.i.. At day 6 p.i., no significant differences concerning the composition of the lymphocyte subpopulations were observed between *Ceacam1^−/−^* and WT mice (Fig. 4B). At day 17 p.i., the proportion of CD3^+^ and CD8^+^ T cells was increased in WT mice compared to *Ceacam1^−/−^* mice whereas the proportion of CD19^+^ and CD4^+^ cells was decreased. This was even more pronounced at day 42 p.i. (Fig. 4B). In both mouse lines, the proportion of CD62L^high^ T cells strongly decreased during infection. However, the decrease was significantly stronger in WT mice than in *Ceacam1^−/−^* mice (Fig. 4B). The absolute cell numbers of the lymphocyte subpopulations (calculated using total splenocyte numbers and the proportions of the subpopulation) revealed that all lymphocyte subpopulations with the exception of CD62L^high^ T cells (where absolute cell numbers actually decreased in WT mice) increased more strongly in *Ceacam1^−/−^* mice than in WT mice between day 6 and day 17 p.i., with the difference being most prominent for B cells and least pronounced for CD8^+^ T cells (Fig. 4C). At day 42 post infection, the numbers of B cells and CD4^+^ T cells were still higher in *Ceacam1^−/−^* mice than in WT mice, while the number of CD8^+^ T cells was nearly identical in both mouse strains (Fig. 4C). Interestingly, while WT mice had almost no naïve (CD62L^high^) T cells at day 42 p. i., *Ceacam1^−/−^* mice displayed naïve T cell numbers comparable to the level at day 6 p.i. (Fig. 4C).

### The frequency of virus-specific CD8^+^ T cells is significantly reduced in *Ceacam1^−/−^* mice

**Figure 5:**
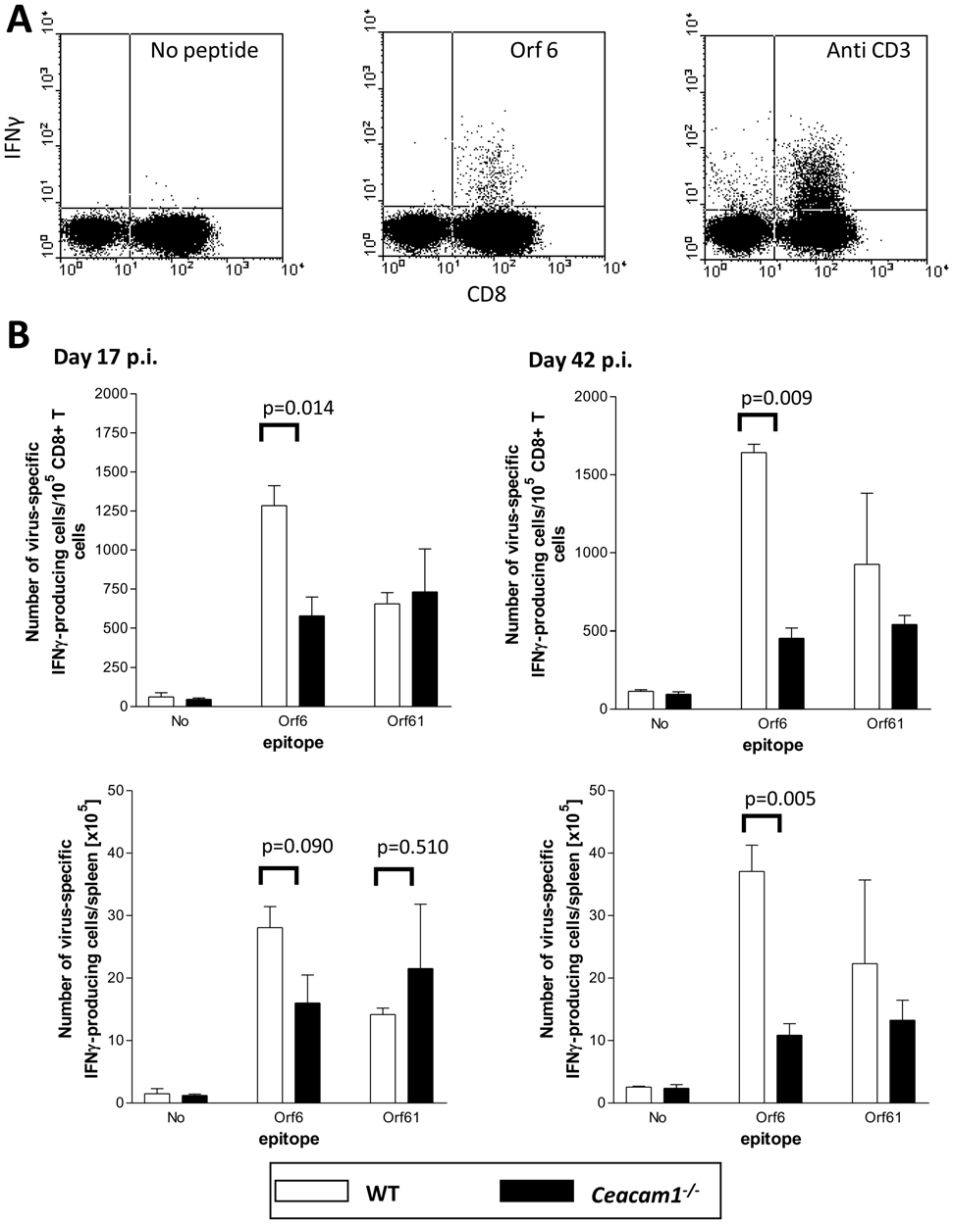
*Ceacam1^−/−^* mice develop a significant weaker MHV-68-specific CD8^+^ T cell immune response during infection than WT mice. Mice were infected i.n. with 5×10^4^ PFU of MHV-68. Spleen cells (3×10^6^/mL) obtained on days 17 and 42 p.i. were restimulated with MHV-68 ORF6- and ORF61-deduced peptides prior to intracellular staining for IFN-γ. Double-positive (CD8^+^ IFN-γ^+^) cells were peptide-specific T cells. A) Dotplots of a representative experiment (ORF6 at day 17) with controls (without addition of peptides or after stimulation with anti-CD3 mAb). The mean numbers of double-positive CD8^+^ IFN-γ^+^ T cells per 10^5^ CD8^+^ spleen cells±SD of three individual mice (upper panel) and the mean number of double-positive CD8^+^ IFN-γ^+^ T cells per spleen±SD of three individual mice (lower panel) are shown. The p values for the differences between *Ceacam1^−/−^* and WT mice are indicated.

To investigate the virus-specific T cell response, we determined the frequency of IFN-γ-producing CD8^+^ T cells in the spleens of infected mice upon *in vitro* restimulation with epitope-specific peptides (Fig. 5). Two epitopes were chosen which exhibit a different expression pattern during virus infection. ORF6 is expressed only during lytic infection while ORF61 is mainly expressed in the spleen during establishment of latency [34]–[36]. As shown in Fig. 5B (upper panel), the frequency of ORF6-specific IFN-γ-producing CD8^+^ T cells was significantly lower in *Ceacam1^−/−^* mice, as compared to WT mice, both on days 17 and 42 p.i.. The lower frequency of ORF6-specific CD8^+^ T cells in *Ceacam1^−/−^* mice was partially compensated by their higher total number on day 17 but not on day 42 p.i. (Fig. 5B, lower panel). There was also a reduced number of ORF61-specific IFN-γ-producing CD8^+^ T cells at day 42 p.i. in *Ceacam1^−/−^* mice. However, this difference did not reach statistical significance. Taken together, the extent of the early virus-specific T cell response is similar in the two mouse strains but the duration or maintenance of the virus-specific T cell response is reduced in *Ceacam1^−/−^* mice, when compared to WT mice.

### Ceacam1^−/−^ mice show a stronger virus-specific antibody response than WT mice

**Figure 6:**
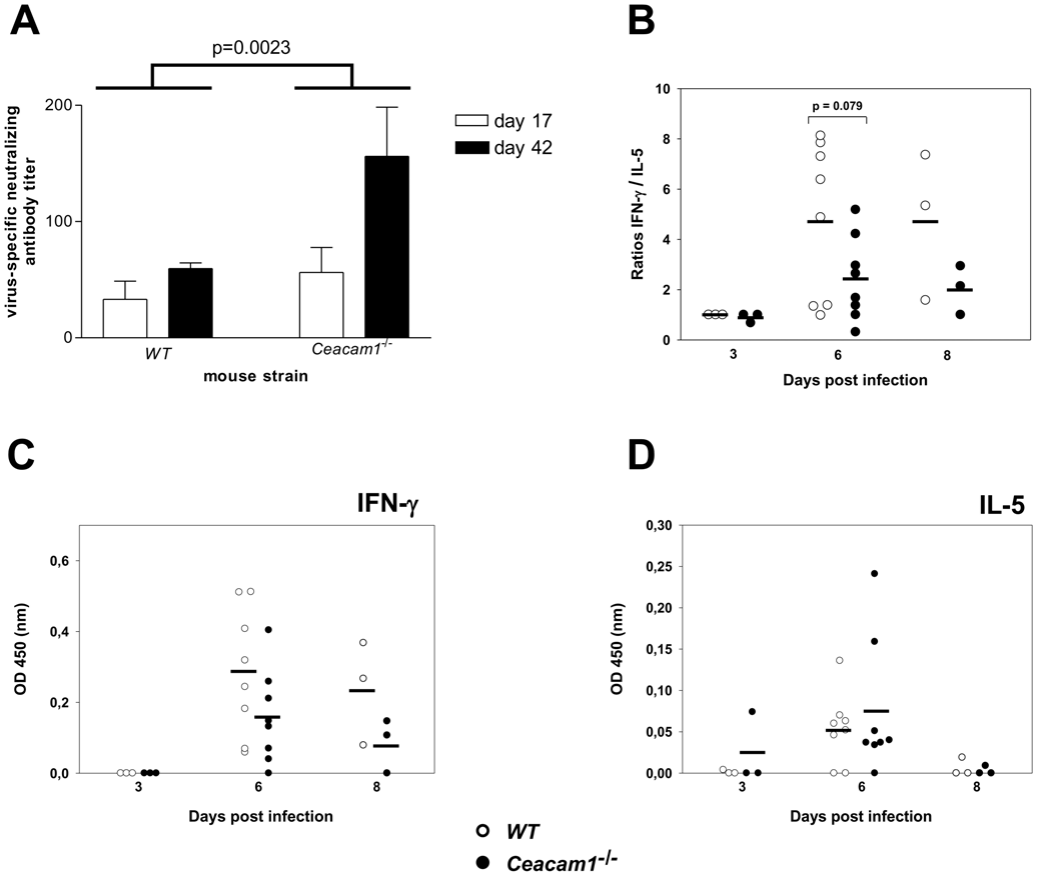
*Ceacam1^−/−^* mice show stronger virus-specific antibody responses and exhibit lower IFN-γ but higher IL-5 serum levels in comparison to WT mice. A) Mice were infected i.n. with 5×10^4^ PFU of MHV-68. At days 17 and 42 after infection, blood was collected and serum was prepared and used in a virus neutralization assay. Data shown are means±SD compiled from two to four independent experiments. In each experiment, sera from 3 to 5 mice per group were pooled. Statistical analysis was performed using two-way ANOVA. The differences of the antibody levels were statistically significant between the two mouse strains, as indicated in the figure, as well as between the two time points. B) Mice were infected i.n. with 5×10^4^ PFU. At days 3, 6 and 8 after infection, cytokine levels in sera were determined by ELISA. Ratios were calculated on the basis of the raw data shown in C and D. Each symbol represents a single mouse. The horizontal bars indicate the means.

To investigate the humoral immune response, we analyzed the virus-specific antibody levels in sera of infected mice. At days 6, 17 and 42 after infection, serum was prepared from blood and used in a virus neutralization assay. At day 6 after infection, the levels of virus-specific antibodies in *Ceacam1^−/−^* and WT mice were below the detection limit of the assay (data not shown). Virus-specific antibodies were detectable at day 17 p.i. and their amount further increased until day 42 p.i. in both mouse groups. The levels of virus-specific antibodies were significantly higher in *Ceacam1^−/−^* mice compared to WT mice, as determined by two-way analysis of variance (ANOVA) (Fig. 6A).

### The immune response in *Ceacam1^−/−^* mice shows a trend towards lower IFN-γ but higher IL-5 levels

The weaker virus-specific T cell but stronger humoral immune response of *Ceacam1^−/−^* mice, when compared to WT mice, suggested that the immune response of *Ceacam1^−/−^* mice might be shifted towards a type2 response. To test this hypothesis, we analyzed the levels of IFN-γ and IL-5 in sera of infected mice at different time points after infection. Although there was considerable variation between individual mice in both groups, we indeed observed a trend towards lower IFN-γ but higher IL-5 levels in sera of infected *Ceacam1^−/−^* mice, when compared to infected WT mice, as reflected by the lower IFN-γ to IL-5 ratios in *Ceacam1^−/−^* mice at days 6 and 8 after infection (Fig. 6B).

### The expression of CEACAM1 increases after infection of WT mice with MHV-68

**Figure 7:**
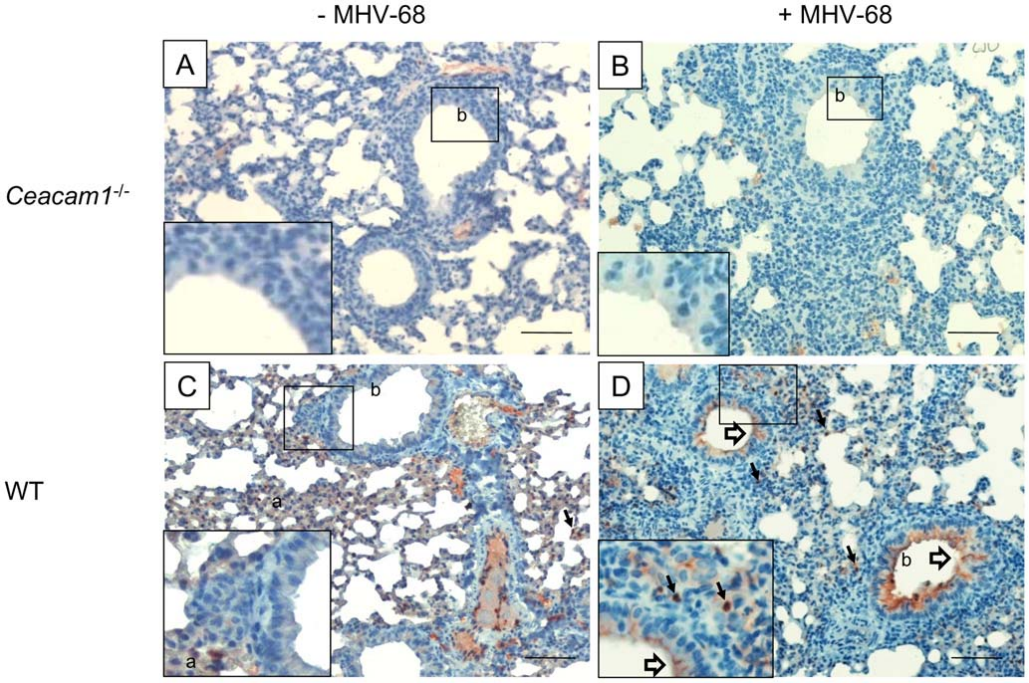
Upregulation of CEACAM1 in lungs of infected WT mice. CEACAM1 expression was visualized in lung sections by staining with mAb CC1, followed by peroxidase-conjugated secondary antibody. CEACAM1 was neither expressed in uninfected *Ceacam1^−/−^* mice (A, insert in A) nor in MHV-68-infected *Ceacam1^−/−^* mice (B, insert in B). In uninfected WT mice, CEACAM1 was readily detectable on immune cells (solid arrows) and on alveolar cells but not or only marginally on bronchial epithelial cells (open arrows) (C). After MHV-68 infection, airway epithelial cells strongly upregulated CEACAM1 (D, arrows and insert in D). Bars, 200 µm (A-D), 50 µm (insert in A-D). a, aveolar cells; b, bronchial epithelial cells.

The finding that *Ceacam1^−/−^* mice displayed a reduced virus-specific T cell response (Fig. 5), yet controlled the acute lung infection more efficiently than WT mice (Fig. 1), as well as the observation that CEACAM1 can inhibit NK cell cytotoxicity when co-ligated with NK cell-activating receptors [31], prompted us to investigate the expression of CEACAM1 in the lungs of *Ceacam1^−/−^* and WT mice by immunohistochemistry. As expected, no CEACAM1 expression could be detected in lungs of uninfected and infected *Ceacam1^−/−^* mice (Fig. 7A and B). In contrast, CEACAM1 expression was readily detectable in uninfected WT mice and was upregulated after infection, especially in bronchial epithelial cells (Fig. 7C and D).

## Discussion

In this study, we investigated the role of the inhibitory molecule CEACAM1 in the immune response to MHV-68 infection using *Ceacam1^−/−^* mice. When compared to WT mice, *Ceacam1^−/−^* mice had lower virus titers in the lungs during acute lytic replication. At day 17 p.i., *Ceacam1^−/−^* mice displayed increased splenomegaly and a higher latent viral load in the spleen as determined both by *ex vivo* reactivation assay and quantitative PCR. At day 42 p.i., *Ceacam1^−/−^* mice still displayed increased splenomegaly. Disparities in the immune response to MHV-68 infection were also observed. At days 17 and 42 p.i., *Ceacam1^−/−^* mice displayed, when compared to WT mice, an aberrant lymphocyte subset composition in the spleen and a reduced frequency of MHV-68-specific CD8^+^ T cells. In contrast, higher levels of virus-specific antibodies were observed in *Ceacam1^−/−^* mice. In addition, *Ceacam1^−/−^* mice showed a cytokine profile suggestive for a shift towards a Th2 type immune response.

So far, no reports investigating the role of inhibitory receptors during MHV-68 infection are available. However, using MHV-68, the role of several co-stimulatory molecules in the control of and the immune response to acute and chronic gammaherpesvirus infections has been investigated [37]. Both CD40 and CD40L have been shown to be dispensable for the control of acute infections but to be important for the long-term control of MHV-68 [38]–[40]. CD28 is required for an effective humoral immune response to MHV-68 but not for acute or long-term T cell responses, and *CD28^−/−^* mice can control the virus [39], [41]. CD80 and CD86 single-knockout mice are able to control MHV-68 infection, while CD80/CD86 double-knockout mice fail to maintain efficient long-term control of MHV-68, demonstrating that these molecules play overlapping roles in the control of MHV-68 [42], [43]. In *CD80^−/−^/CD86^−/−^* mice, primary antiviral CD8^+^ T cell responses and the induction of neutralizing antibodies are impaired [42], [43]. The 4-1BBL/4-1BB costimulatory pathway was recently found to be important for the maintenance of antiviral CD8^+^ T cell function during latent MHV-68 infection [44].

Compared to the co-stimulatory molecules CD28, CD40, CD40L, CD80/CD86 and 4-1BBL, CEACAM1 seems to play a different role during MHV-68 infection (Table 1). During acute replication in the lungs, the absence of CEACAM1 resulted in lower viral titers. We hypothesize that this may be due to an increased activity of NK cells since it has been shown that CEACAM1 can inhibit NK cell cytotoxicity when co-ligated with NK cell-activating receptors [31]. In WT mice, lung epithelial cells indeed upregulated CEACAM1 early during infection (Fig. 7C and D), suggesting that the absence of CEACAM1 in *Ceacam1^−/−^* mice promotes NK cell activity while in WT mice, the strong expression of CEACAM1 downregulates NK cell activity. Expression of CEACAM1 by NK cells, in particular after activation, has been shown by others [45]. Clearly, much work needs to be done to confirm our hypothesis. It would be remarkable if in *Ceacam1^−/−^* mice, NK cells would contribute to the clearance of lytic virus since this would be different to the situation in other strains of mice where it has been reported that NK cells are not involved in gammaherpesvirus clearance [46], [47].

**Table 1:** Role of co-stimulatory and inhibitory receptors during MHV-68 infection.

Mouse strain	Acute viral load (lung)	Latent viral load (spleen) early/late	Spleno-megaly	Viral re-activation (lung)	References
CD40 -/-	=	↓/ =	↓	yes	37,39,40,57
CD40L -/-	=	↓/ =	↓	yes	37,38
CD28 -/-	= or ↑	= / =	=	no	37,39,41,43
CD80 -/-	=	= / =	=	no	42,43
CD86 -/-	=	= / =	=	no	42,43
CD80/86 -/-	=	= / =	=	yes	42,43
4-1BBL -/-	=	= /↑	=	no	44
					
CEACAM1 -/-	↓	↑/ =	↑	no	this study

= , similar; ↑, increased; ↓, decreased (compared to wildtype mice).

The absence of CD28, CD40, CD40L, CD80/CD86 or 4-1BBL did either not affect viral replication in the early phase of infection or early viral titers were rather increased [38], [39], [41]–[44]. In addition, in the absence of CD40, CD40L or CD80/CD86, viral reactivation in the lungs was observed at later time points after infection. In contrast, we did not detect viral reactivation in the lungs of *Ceacam1^−/−^* mice 42 days after infection. While we detected increased splenomegaly and a higher splenic latent viral load in *Ceacam1^−/−^* mice at day 17 after infection, a similar genomic load in the spleens of WT and *Ceacam1^−/−^* mice was detected at days 42 and 300 after infection. This again is contrary to MHV-68 infections in the absence of CD40, CD40L or CD80/CD86, where splenomegaly and/or viral load during latency amplification were either similar or lower [38], [39], [41]–[43]. The overall higher genomic load during early latency around day 17 in *Ceacam1^−/−^* mice was not only due to a higher total number of cells as well as B cell numbers but also to an increased frequency of latently infected cells, the existence of the latter most likely being the consequence of the reduced frequency of MHV-68-specific CD8^+^ T cells.

With regard to the immune response to MHV-68, we also noted a number of differences between the role of CD28, CD40, CD40L and CD80/CD86, and that of CEACAM1. We observed an aberrant lymphocyte subset composition in the spleen of MHV-68 infected *Ceacam1^−/−^* mice, while the lymphocyte subset distribution was unaltered in mice lacking CD28 or CD80/CD86 [39], [43]. The absence of CEACAM1 resulted in a reduced frequency of MHV-68-specific CD8^+^ T cells. For mice lacking CD80/CD86, one study reported an impaired primary expansion of MHV-68-specific CTLs [42], while in another study, the frequency of MHV-68-specific CTLs was unaffected by the lack of CD80/CD86 [43]. In the absence of CD28 or CD40L, the frequency of MHV-68-specific CTLs was unaltered [38], [39], [41]. Furthermore, the absence of CD28, CD40 or CD80/CD86 resulted in a reduced production of MHV-68-specific antibodies [37], [39], [41]–[43], whereas we observed higher levels of virus-specific antibodies in *Ceacam1^−/−^* mice.

Taken together, the results obtained in *Ceacam1^−/−^* mice support the view that a main function of CEACAM1 expressed by lymphocytes is to regulate their proliferation. Thus, the absence of CEACAM1 resulted in a more severe splenomegaly which could be due to increased proliferation and/or enhanced recruitment of cells. Indeed, the abnormal increase in absolute numbers of certain lymphocytes correlated with the amount of CEACAM1 on lymphocytes of the different subpopulations. B cells which constitutively express CEACAM1 were most abundant in *Ceacam1^−/−^* mice after infection. In contrast, CD8^+^ T cells which express CEACAM1 only for a certain time span upon activation [48] increased much less. Greicius and coworkers reported that CEACAM1-mediated signals, induced by cross-linking with anti-CEACAM1 antibodies, facilitate B cell proliferation and antibody production [24]. Whether this apparent discrepancy is due to differences of *in vitro* as opposed to *in vivo* B cell stimulation or to additional regulatory cells present *in vivo* has to be elucidated. The different effects of CEACAM1-mediated growth control on B versus T cells may lead to a bias of the immune response. Alternatively, signaling through CEACAM1 may directly regulate the bias of the immune response by facilitating a Th1 immune response. This is supported by our previous observation that CEACAM1 signaling in murine DC facilitates MHC-II-restricted priming of naïve CD4^+^ T cells with a Th1-biased polarization [25]. Therefore, in the absence of CEACAM1, a type2 immune response might be favored, as indicated by the higher titer of virus-specific antibodies, the lower level of virus-specific CD8^+^ T cells and the trend towards a decreased IFN-γ to IL-5 ratio.

Since we analyzed the consequences of the complete absence of CEACAM1 isoforms on the course of a gammaherpesvirus infection, we cannot attribute the observed effects to a specific isoform of CEACAM1. However, the fact that CEACAM1-L exceeds the levels of CEACAM1-S in immune cells [49], and that the inhibitory function of CEACAM1-L overrules the function of the short isoform [32], indicates that the alterations of the immune response are mainly due to the absence of CEACAM1-L.

What is the underlying mechanism of the reduced virus-specific T cell response in *Ceacam1^−/−^* mice during the latent phase of MHV-68 infection? One possibility is that the negative signal provided by CEACAM1 during the early phase of the immune response may prevent an overwhelming T cell activation in WT mice. Loss of CEACAM1, therefore, could result in exhausted T cells that cannot further control chronic virus infection [50]. Our experiments which identified CD8^+^ T cell responses to an “early” and a “late” T cell epitope suggest that the survival time of T cells which are activated in the absence of a co-inhibitory molecule may be shorter and, hence, the control of long lasting infections may be impaired.

In summary, due to an altered immune response, *Ceacam1^−/−^* mice displayed an enhanced control of the acute lytic MHV-68 lung infection but elevated splenic viral loads and increased splenomegaly during latency amplification. Therefore, interference with CEACAM1 function during gammaherpesvirus infections of humans, perhaps in combination with interference with other inhibitory receptors, might have clinical value in the future.

## Materials and Methods

### Cell lines and virus stocks

BHK-21 cells were grown in Glasgow-MEM (Biochrom AG, Berlin, Germany) supplemented with 5% fetal calf serum (FCS), 5% tryptose phosphate broth, 2 mM L-glutamine, 100 U/mL penicillin and 100 µg/mL streptomycin. NIH3T3 cells were grown in DMEM High Glucose (Cell Concepts, Umkirch, Germany) supplemented with 10% FCS, 2 mM L-glutamine, 100 U/mL penicillin and 100 µg/mL streptomycin. The original stock of MHV-68 (clone G2.4) was obtained from Drs. J. Stewart and A. Nash (University of Edinburgh, Edinburgh, UK). Working stocks of virus were prepared as previously described [51]. Briefly, stocks were grown by infecting BHK-21 cells. After showing complete cytopathic effect (CPE), BHK-21 cells were harvested and the supernatant was used as working stock after two times freezing/thawing the cells and removing cell debris by centrifugation. Virus titers were determined by plaque assays. Briefly, 10-fold dilutions were incubated on BHK-21 cells for 90 min at 37°C. After removing the inoculum, cells were incubated for 5 days at 37°C with fresh medium containing methylcellulose. Cells were stained with 0.1% crystal violet solution to determine the number of plaques.

### In vivo experiments

*Ceacam1^−/−^* mice and WT littermates were generated as described previously [52] and bred under standard pathogen-free conditions in the animal facility of the Helmholtz Zentrum München. Anesthetized mice were infected i.n. with 5×10^4^ plaque forming units (PFU) of MHV-68. To determine lytic virus titers, the lungs were harvested on various days after i.n. infection and homogenized. After two times freezing and thawing the homogenates, plaque assays were performed with 10-fold dilutions of the supernatants on BHK-21 cells. *In vitro* amplification of viral titers was performed as described by others [42]. To determine the frequency of cells carrying virus reactivating from latency, spleens were harvested, single splenocyte suspensions were prepared and analyzed in an ex vivo limiting dilution reactivation assay as described [53]. Briefly, serial threefold dilutions of infected mouse splenocytes were plated on monolayers of 1×10^4^ low-passage NIH3T3 cells per well in 96-well tissue culture plates. 24 wells were plated per dilution (starting with 1,5×10^5^ cells/well). NIH3T3 cells were screened microscopically for a viral cytopathic effect (cpE) for up to 3 weeks. To differentiate between latently infected cells and infectious virus in the samples, serial threefold dilutions of spleen cells were plated before or after mechanical disruption of viable cells (by two freeze-thaw cycles). No infectious virus was detected in samples of mechanically disrupted cells (data not shown). Frequencies of reactivating cells were calculated on the basis of the Poisson distribution by determining the cell number at which 63.2% of the wells scored positive for CPE. All animal experiments were in compliance with protocols approved by the local Animal Care and Use Committee.

### FACS analysis

For surface staining, cells were suspended in PBS/0.3% w/v BSA supplemented with 0.1% w/v sodium azide. Nonspecific binding of antibodies to Fc receptor was blocked by preincubating cells with 1 µg anti-CD16/CD32 mAb 2.4G2/10^6^ cells. Cells were incubated with 0.5 µg/10^6^ cells of the relevant mAb for 30 min at 4°C, washed twice, and subsequently incubated with a second-step reagent for 15 min at 4°C. Cells were washed twice, fixed with 2% paraformaldehyde for 1 h and analyzed on a FACScan (Becton & Dickinson, Mountain View, CA). The following reagents and mAbs from Pharmingen were used: FITC-conjugated and APC-conjugated anti-CD3; FITC-conjugated and PE-conjugated anti-CD4; FITC-conjugated and PE-conjugated anti-CD8; FITC-conjugated and PE-conjugated anti-CD11c; PE-conjugated and allophycocyanin (APC)-conjugated anti-CD19; PE-conjugated anti-CD25; PE-conjugated anti-CD62L; PE-conjugated anti-CD69; APC-conjugated anti-NK-1.1; FITC-conjugated anti-GR-1. Furthermore, we used FITC-conjugated IgG1 mAb R3-34, PE-conjugated IgG1 mAb R3-34. Expression of CEACAM1 was analysed using mAb CC1 (provided by Kathryn V. Holmes, Aurora, CO, USA) and FITC-conjugated and PE-conjugated rat anti-mouse IgG1 mAb (A85-1).

### Intracellular cytokine staining assay

Spleen cells (10^6^/mL) were incubated for 60 min in RPMI medium with 5 µg/mL ORF61_524-531_/H2-K^b^ (TSINFVKI) or ORF6_487–495_/H2-D^b^ (AGPHNDMEI) peptide (GENOVAC GmbH, Freiburg, Germany) in round bottom 96-well plates. Thereafter, 5 µg/mL brefeldin A (BFA) (Sigma) was added, and the cultures were incubated for another 4 h. Cells were harvested and surface-stained with PE-conjugated anti-CD8 mAbs (Pharmingen). Surface-stained cells were fixed with 2% paraformaldehyde in PBS before intracellular staining for IFN-γ was performed. Fixed cells were resuspended in permeabilization buffer (HBSS, 0.5% BSA, 0.5% saponin, 0.05% sodium azide) and incubated with FITC-conjugated anti-IFN-γ mAb (Pharmingen) for 30 min at RT and washed twice in permeabilization buffer. Stained cells were resuspended in PBS/0.3% w/v BSA supplemented with 0.1% w/v sodium azide and analyzed by flow cytometry. The number of IFN-γ^+^ cells per 10^5^ CD8^+^ cells was determined. For control of antigen-independent IFN-γ production we did not add any peptide to spleen cells, since previous experiments showed that this control is equivalent to the addition of H2-K^b^-restricted peptides of irrelevant antigens.

### Measurement of virus-specific antibody responses

Virus-specific antibodies in sera of infected mice were determined by virus neutralization assays. Eight twofold dilutions of serum were made, starting at a 1/20 dilution. The diluted serum samples were mixed with 50 PFU of MHV-68 and incubated on ice for 1 h. One hundred µL of each dilution were then added in quadruplicate to NIH3T3 cells in 96 well plates and incubated at 37°C. After 4 to 5 days, the wells were scored for CPE. The serum neutralization titer was calculated by the method of Reed and Muench [54].

### Measurement of latent viral load by quantitative real time PCR

Latent viral load in the spleens of infected mice was quantified by real-time PCR using the ABI 7300 Real Time PCR System (Applied Biosystems, Foster City, CA, USA). Amplification was performed with Taqman universal PCR master mix and universal cycling conditions (Applied Biosystems). Quantification of viral DNA copy number was achieved by amplification of a 70 bp region of the MHV-68 gB gene using primers and probes as described by others [55]. A standard curve was generated using known amounts of a plasmid containing the *Hind*III-N fragment of MHV-68 (containing the gB gene). DNA was extracted from spleen cells using the QIAamp DNA Mini Kit (Qiagen, Hilden, Germany) and quantified by UV spectrophotometry. One hundred ng of DNA were used per reaction. Although the assay was able to detect less than 10 viral genomes per sample as determined by spiking DNA from the spleen of an uninfected mouse with a graded number of copies of the plasmid containing the *Hind*III-N fragment of MHV-68, constant quantification was possible at 50 or more copies per sample. Thus, in accordance with published recommendations [56], the quantification limit was set at 50 copies per sample. For statistical analysis, samples with a calculated copy number below 50 were assigned 50 copies. A fragment of the murine ribosomal protein L8 gene (*Rpl8*) was amplified using the Taqman gene expression assay (Assay ID: Mm00657299_g1) (Applied Biosystems) and used to normalize for input DNA between samples. A standard curve for Rpl8 was constructed by serial dilution of a plasmid containing Rpl8 (RZPD clone IRAVp968B01123D6) (RZPD, Berlin, Germany). The data are presented as viral genome copy numbers relative to the gene copy numbers of *Rpl8*.

### Measurement of cytokines

Levels of IFN-γ and IL-5 in sera of mice were determined by ELISA using recombinant cytokine standards and pairs of capture and biotin-conjugated secondary antibodies (Pharmingen, San Diego, CA) according to the instructions of the manufacturer.

### Histopathological analysis of lung tissue

Lungs of mice were harvested on day 6 after i.n. infection and fixed in 10% formalin in PBS. For histopathological analysis, organs were embedded in paraffin. Sections were cut, stained with hematoxylin and eosin (H/E) and analyzed by light microscopy. To visualize expression of CEACAM1, sections were stained with mAb CC1, followed by reagents of mouse-to-mouse IHC Detection kit (Millipore, Billerica, MA).

### Statistical methods

If not otherwise indicated, data were analyzed by two-tailed Student's t-test.
